# Characterization of N-Acetyl Cysteine Adducts with Exogenous and Neutrophil-Derived 2-Chlorofatty Aldehyde

**DOI:** 10.3390/antiox12020504

**Published:** 2023-02-16

**Authors:** Shubha Shakya, Reagan M. McGuffee, David A. Ford

**Affiliations:** 1 Center for Cardiovascular Research, Saint Louis University School of Medicine, St. Louis, MO 63104, USA; 2 Edward A. Doisy Department of Biochemistry and Molecular Biology, Saint Louis University School of Medicine, St. Louis, MO 63104, USA

**Keywords:** myeloperoxidase, chlorinated lipids, N-acetyl cysteine, neutrophils

## Abstract

Hypochlorous acid is produced by leukocyte myeloperoxidase activity. 2-Chlorofatty aldehydes (2-ClFALDs) are formed when hypochlorous acid attacks the plasma membrane phospholipid plasmalogen molecular subclass and are thus produced following leukocyte activation as well as in the lungs of mice exposed to chlorine gas. The biological role of 2-ClFALD is largely unknown. Recently, we used an alkyne analog (2-ClHDyA) of the 2-ClFALD molecular species, 2-chlorohexadecanal (2-ClHDA), to identify proteins covalently modified by 2-ClHDyA in endothelial cells and epithelial cells. Here, we demonstrate that 2-ClHDA reduces the metabolic activity of RAW 264.7 cells in a dose-dependent manner. 2-ClHDyA localizes to the mitochondria, endoplasmic reticulum and Golgi in RAW 264.7 cells and modifies many proteins. The thiol-containing precursor of glutathione, N-acetyl cysteine (NAC), was shown to produce an adduct with 2-ClHDA with the loss of Cl^−^ (HDA–NAC). This adduct was characterized in both positive and negative ion modes using LC-MS/MS and electrospray ionization. NAC treatment of neutrophils reduced the 2-ClFALD levels in PMA-stimulated cells with subsequent increases in HDA–NAC. NAC treatments reduced the 2-ClHDA-elicited loss of metabolic activity in RAW 264.7 cells as well as 2-ClHDA protein modification. These studies demonstrate that 2-ClFALD toxic effects can be reduced by NAC, which reduces protein modification.

## 1. Introduction

2-Chlorofatty aldehydes (2-ClFALDs) are liberated when hypochlorous acid (HOCl) attacks the vinyl ether bond of plasmalogen phospholipids [1]. HOCl is produced in activated neutrophils by myeloperoxidase using hydrogen peroxide (H_2_O_2_) and chloride ion as substrates. Neutrophil-derived 2-ClFALD has been shown to accumulate in activate human neutrophils, human atherosclerotic lesions and infarcted rat myocardium [2,3,4,5]. Recently, the oxidized product of 2-ClFALD, 2-chlorofatty acid (2-ClFA), has been shown to correlate with sepsis mortality [6]. Myeloperoxidase-derived 2-ClFALD and 2-ClFA are suggested to be involved in endothelial dysfunction, neutrophil chemotaxis, NETosis and vascular tone [6,7,8,9]. Additionally, the Ford lab and other labs have shown that 2-ClFALD and 2-ClFA cause cell toxicity [10,11,12,13]. 

2-ClFALD is an electrophile with a carbonyl group and chlorine attached to the alpha carbon. Duerr et al. have shown that 2-ClFALD can form an adduct with glutathione via a nucleophilic substitution reaction [14]. Duerr et al. also showed that 2-ClFALD can modify proteins in a thiol-dependent manner [15]. Additionally, 2-ClHDA can modify proteins via Schiff base formation [10,16]. Recently, we identified proteins modified by 2-ClFALD without Schiff base stabilization in endothelial cells and epithelial cells [17,18]. 2-ClFALD has been shown to cause mitochondrial dysfunction, apoptosis via the activation of caspase 3 and an altered intracellular redox balance in brain microvascular endothelial cells (BMVEC) [10]. Additionally, 2-ClFALD elicits mitochondrial dysfunction in human small airway epithelial cells [17]. 

Here, we demonstrate the reactivity of 2-ClFALD with N-acetyl cysteine (NAC). The NAC adduct with the 2-ClFALD molecular species 2-chlorohexadecanal (2-ClHDA) was characterized by LC-MS/MS. The adduct is produced in the presence of exogenously applied 2-ClHDA as well as endogenously produced 2-ClHDA by activated neutrophils. Furthermore, the reduction of 2-ClHDA levels by NAC reduces the decreases in metabolic activity caused by 2-ClHDA. NAC also reduces 2-ClHDA protein modification in RAW 264.7 cells. 

## 2. Material and Methods

### 2.1. Materials

Cell culture supplies were purchased from Sigma-Aldrich (St. Louis, MO, USA). A Click-It Cell Reaction Buffer Kit was purchased from Thermo Fisher Scientific (Waltham, MA, USA; catalog no. C10269). Rabbit polyclonal anti-calnexin (catalog no. ab22595), anti-cytochrome c oxidase subunit IV (anti-COX IV; catalog no. ab202554), mouse polyclonal anti-Golgi matrix protein 130 (anti-GM130; catalog no. ab169276), goat anti-rabbit IgG H&L (Alexa Fluor^®^ 488) (catalog no: 150077) and goat anti-mouse IgG H&L (Alexa Fluor^®^ 488) (catalog no. ab150113) antibodies were purchased from Abcam (Cambridge, UK). All other chemicals were purchased from Sigma-Aldrich (St. Louis, MO, USA) or Thermo Fisher Scientific (Waltham, MA, USA). The alkyne analog of 2-ClHDA (2-ClHDyA) was synthesized and quantified as described previously [15,18,19,20].

### 2.2. RAW 264.7 Cell Culture and Lipid Treatments

RAW 264.7 cells (ATCC, Manassas, VA, USA, Cat. TIB-71) were grown in Dulbecco’s Modified Eagle medium (DMEM, Sigma-Aldrich D-5796) supplemented with 10% FBS, 1 mM sodium pyruvate and 6 mM L-glutamine in 10% CO_2_/90% air at 37 °C. The RAW 264.7 cells were used through passage 15 for these experiments. The cells were treated with indicated concentrations of ClHDA and ClHDyA in 2% FBS in DMEM.

### 2.3. Detection of 2-ClHDyA by Immunofluorescence

The cells were plated on sterile coverslips in 6-well plates and treated with 10 µM 2-ClHDyA in 2% FBS for 30 min. The cells were washed quickly with PBS and then were fixed with formalin for 10 min. The cells were permeabilized with 0.25% Triton X-100 for 10 min. The cells were washed with 2% (*w*/*v*) BSA in PBS. They were then labeled with 5 μM azide–carboxytetramethylrhodamine (azide–TAMRA) (Sigma-Aldrich; catalog no. 760757) by using the Click-It Cell Reaction Buffer Kit (Thermo Fisher, Waltham, MA, USA; catalog no. C10269) following the manufacturer’s protocols. The click reagents were washed away with 2% BSA in PBS. The cells were then incubated with primary antibodies against COXIV (1:500), GM130 (1:142) and calnexin (1:1000) overnight at 4 °C. The next day, the cells were washed three times with PBS for 5 min to remove any unbound primary antibody. The cells were incubated with the goat anti-mouse IgG secondary antibody (1:500) labeled with Alexa 488 or the goat anti-rabbit IgG secondary antibody (1:500) for 1 h. The coverslips were mounted onto microscope slides with a Vectashield solution containing 4′,6-diamidino-2-phenylindole (DAPI; Vector Laboratories, Newark, CA, USA; catalog no. H1200).

### 2.4. Confocal Microscopy

A Leica SP5 confocal microscope (Leica Microsystems, Mannheim, Germany) with a 63 × 1.4 oil immersion objective was used to acquire the images. A He/Ne laser was used as the source of excitation light at 488 nm for all organelles and at 543 nm for 2-ClHDyA clicked with azide-TAMRA. The fluorescence of organelles was detected between 500 and 540 nm in channel 3. Azide-TAMRA fluorescence was detected between 570 and 650 nm. DAPI fluorescence was excited with an ultraviolet laser at 405 nm in channel 2 and detected between 440 and 470 nm. The Alexa 488 and TAMRA fluorescence signals were acquired simultaneously. 

### 2.5. Cell Metabolic Activity Assessed by 3-(4,5-dimethyl-2-thiazolyl)-2,5-diphenyl-2H-tetrazolium Bromide Assay 

The metabolic activity of the RAW 264.7 cells was examined using the 3-(4,5-dimethyl-2-thiazolyl)-2,5-diphenyl-2H-tetrazolium bromide (MTT) assay following the manufacturer’s protocol. In brief, 20,000 cells per well were plated in a clear 96-well plate for 20 h. The cells were treated with the indicated concentrations of lipids in 2% FBS in DMEM for the indicated time at 37 °C. After treatment, the medium was replaced with fresh PBS. MTT (Sigma-Aldrich, M2128; 1.2 mM in 100 µL) was added to the cells for 2 h. Seventy-five microliters of medium were removed and 50 µL of DMSO was added and incubated for 10 min at 37 °C. Absorbance was taken at 540 nm on an Enspire multimode plate reader and corrected for background absorbance. Triton-X (0.1%) in the PBS was used as a positive control. The MTT reduction is expressed as percent MTT reduction (vehicle-treated sample designated as 100%).

### 2.6. Sequestering 2-ClHDA with NAC

One milliliter of 2% FBS in DMEM was treated with 50 µM 2-ClHDA and different concentrations of NAC for 1 h at 37 °C. The unreacted 2-ClHDA was extracted as previously described [21]. Lipid extraction of 100 µL of the sample was performed in the presence of 2-chloro-[7,7,8,8-d_4_]hexadecanal by a modified Bligh and Dyer procedure [22] and the 2-ClFALD molecular species were quantified as previously described [23,24].

### 2.7. Human Neutrophil Studies

The human neutrophils were prepared from whole blood using a Ficoll-Hypaque gradient as previously described [3]. These studies were approved and authorized by the Saint Louis University Institutional Review Board Protocol 9952. Informed consent was obtained from the human subjects. Isolated neutrophils were suspended in HBSS (2 million neutrophils per ml). The neutrophils were treated with PMA (200 nM) in ethanol (0.1%) or a vehicle at 37 °C. An amount of 12 mM NAC was added either 30 min before the PMA/vehicle treatment or 25 min after the PMA/vehicle treatment. The incubations were terminated by the addition of 10 mM NEM and subsequent snap freezing. Lipid extraction was performed in the presence of 2-chloro-[7,7,8,8-d_4_]hexadecanal by a modified Bligh and Dyer procedure [22] and the 2-ClFALD molecular species were quantified as previously described [23,24].

### 2.8. Detection of Protein Modification by Lipid

Confluent RAW 264.7 cells in six-well plates were pretreated with 250 µM NAC for 30 min at 37 °C and then were incubated with 2-ClHDyA (25 µM) for 1 h at 37 °C. After the treatment was complete, the cells were lysed with a RIPA buffer containing protease inhibitors (complete mini EDTA-free protease inhibitor cocktail and 400 µM phenylmethylsulfonyl fluoride (PMSF)). The DNA was sheared by passing through a 26 G needle 4–5 times. The insoluble material in the lysates was removed by centrifugation (14 kG for 20 min). The BCA protein assay (Pierce cat. 23225) was used to determine the protein concentration. A click reaction was performed as described previously to conjugate 2-ClHDyA with TAMRA fluorophore [15]. The clicked protein was purified by the methanol chloroform precipitation method. Clicked proteins were resolved on 12% Bis-Tris gels in the stated quantities. After electrophoresis, the gels were sequentially rinsed in water, immediately visualized at 532/580 nm (excitation/emission) and stained with Coomassie blue.

### 2.9. 2-ClHDA and NAC In Vitro Reaction Products and Purification

The reactions were executed in 70 % ethanol in PBS (pH 7). Briefly, 2-ClHDA (0.5 μmol) was first dissolved in 105 µL of ethanol. Then, NAC (5 µmol) in 45 µL PBS (pH adjusted to 7) was added to the 2-ClHDA and incubated for 2 h at 37 °C. The reaction products were resolved on 40 Å silica gel TLC plates with solvents comprised of chloroform–acetone–methanol–water–acetic acid (6:8:2:2:1 *v*/*v*/*v*/*v*/*v*). The TLC plates were visualized with a phosphomolybdic acid stain. The 2-ClHDA–NAC adduct (HDA–NAC) was purified using a Strata-X column, a reversed-phase functionalized polymeric sorbent (Phenomenex, 00M-S033-B0-CB). The eluted adduct was dried under nitrogen and resuspended in methanol.

### 2.10. ESI/MS/MS Characterization of 2-ClHDA and NAC In Vitro Reaction Products

HDA–NAC was diluted and analyzed by ESI/MS/MS (a Thermo Fisher TSQ Quantum Ultra mass spectrometer (Thermo Fisher, Waltham, MA) and XCalibur software (Thermo Fisher) by direct infusion at a flow rate of 5 μL/min. For ESI/MS/MS, the ionization energy and temperature were set at 3700 V and 270 °C for the positive ion mode and 2600 V and 270 °C for the negative ion mode. A collision energy of 15 eV and collision gas of 1.0 Torr Argon were used for the MS/MS analyses in both the positive and negative ion modes.

### 2.11. Extraction and Quantification of HDA–GSH and HDA–NAC

HDA–GSH and HDA–NAC were extracted as described previously [14]. Briefly, for a 1 mL neutrophil suspension, 45 fmol of [d_4_]HDA–GSH was used as the internal standard. The adducts were analyzed by LC/MS/MS. The SRM of 402.17→342.75 was used to detect HDA–NAC.

### 2.12. Statistical Analyses 

GraphPad Prism 8 was used for the statistical analysis. A one-way ANOVA was used to compare the test groups with the control group. Post-hoc analyses are indicated for each study. 

## 3. Results

### 3.1. Effect of 2-ClHDA on Cell Metabolic Activity 

**Figure 1:**
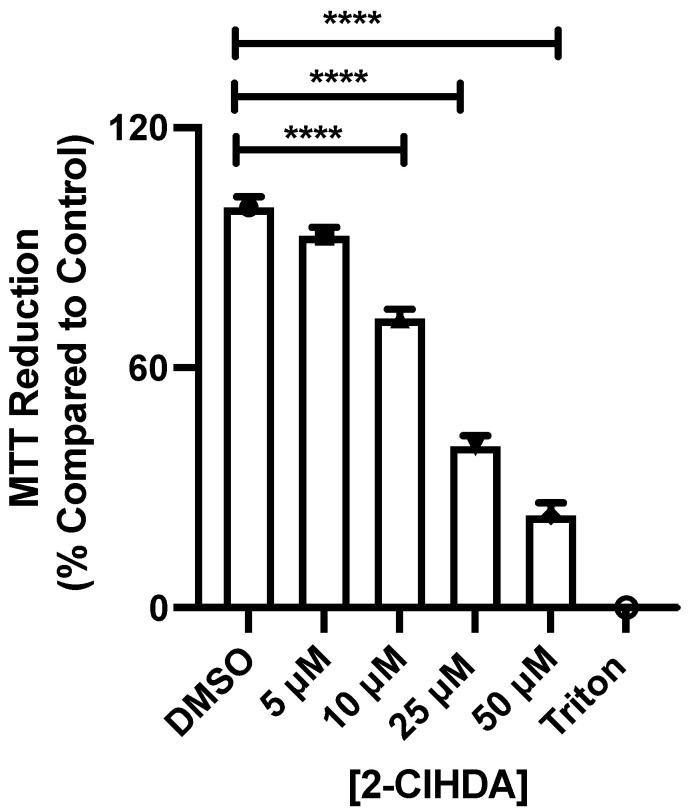
2-ClFALD impairs the metabolic activity of RAW 264.7 cells. RAW 264.7 cells were treated with indicated concentrations of 2-ClHDA for 5 h. Metabolic activity was analyzed by the MTT assay. Results are expressed in % compared to control. Data represent mean values ± SEM of three independent experiments. **** indicates *p* < 0.0001 for indicated comparisons determined by ANOVA with Dunnett’s post-hoc test.

Previous studies have shown that the metabolite of 2-ClFALD, 2-ClFA, leads to the apoptosis of RAW 264.7 cells and primary monocytes through increased ROS production and ER stress [13]. 2-ClFALD has been shown to have toxic effects on brain microvascular endothelial cells [11]. To investigate if the 2-ClFALD molecular species, 2-ClHDA, also caused changes in RAW 264.7 cells, we examined the effect of 2-ClHDA on metabolic activity using the MTT assay within the physiological range of 2-ClHDA previously determined in activated neutrophils [3]. The data shown in Figure 1 reveal that concentrations of as low as 10 µM 2-ClHDA incubated for 5 h significantly decreased the metabolic activity of RAW 264.7 cells. 

### 3.2. Subcellular Localization of 2-ClHDyA 

**Figure 2:**
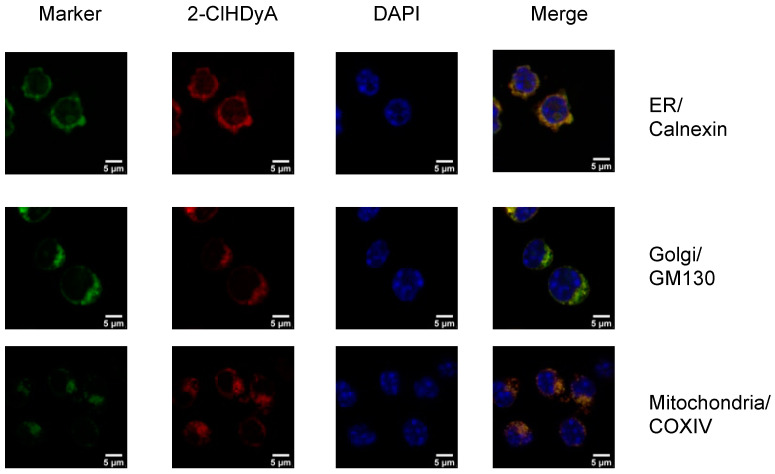
Subcellular localization of 2-ClHDyA in RAW 264.7 at 30 min. Cells were grown to confluence on sterile coverslips and treated with 10 μM 2-ClHDyA for 30 min. Cells were fixed with formalin, permeabilized with Triton X-100 and clicked with azide TAMRA (red). Cells were incubated with primary antibodies against GM130 (Golgi), calnexin (ER) and COXIV (mitochondria), then labeled with Alexa 488 labeled secondary antibodies (green). Cells were mounted in a DAPI-containing solution (blue) and imaged with a Leica SP5 microscope. All fluorescence was taken simultaneously.

Because previous data suggest that 2-ClHDyA modifies proteins in THP-1 cells, human endothelial cells (hCMEC/D3 and EA.hy926 cells), human lung microvascular endothelial cells (HLMVEC), human small airway epithelial cells and mouse HL-1 cardiomyocytes [10,15,17,18,25], we investigated if we could detect the subcellular localization of proteins modified by 2-ClHDyA in RAW 264.7 cells. We treated RAW 264.7 cells with 2-ClHDyA and used click chemistry to conjugate 2-ClHDyA with TAMRA fluorophore. 2-ClHDyA colocalized to ER, Golgi and mitochondria as indicated by calnexin, GM130 and COXIV, respectively (Figure 2). A diffuse pattern for 2-ClHDyA was also observed, suggesting the modification of cytoplasmic proteins. Comparing these data to the metabolic activity reduction elicited by 10 μM suggests that 2-ClHDyA associated with mitochondria may mediate metabolic activity, albeit MTT measurements with 2-ClHDyA were not determined during these short incubation intervals (30 min) employed in these subcellular localization studies. It should be noted that 2-ClHDyA and 2-ClHDA have similar effects on metabolic activity in several cell lines [10,26].

### 3.3. N-Acetyl Cysteine Quenches 2-ClFALD

**Figure 3:**
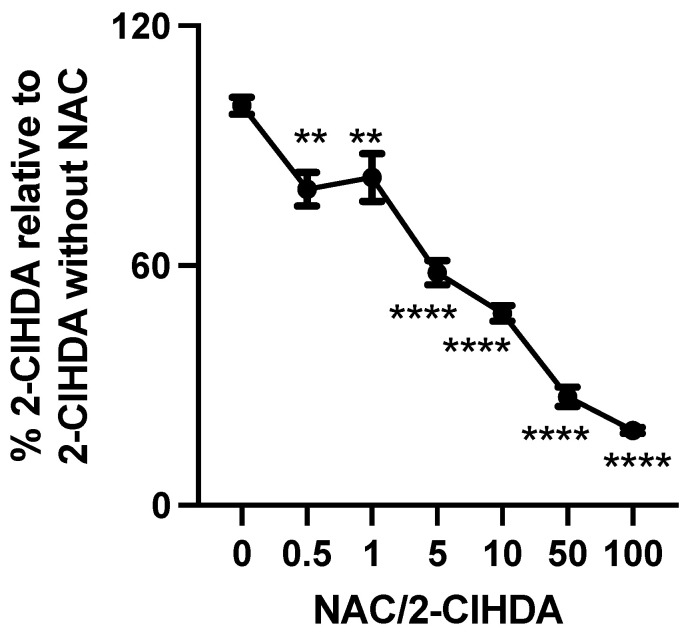
NAC quenches 2-ClHDA. 2% FBS in DMEM spiked with 50 µM 2-ClHDA was treated with indicated concentrations of NAC for 1 h. After 1 h, 2-ClHDA levels were determined as described in “Materials and Methods”. *n* = 3 for each treatment from independent experiments. ** *p* < 0.01, **** *p* < 0.0001 for comparison of NAC treatment with no treatment determined by ANOVA and Dunnett’s post-hoc test.

Aldini et al. [27] have suggested that scavenging reactive carbonyl species, such as 4-hydroxynonenal (HNE), is among the promising approaches to inhibiting protein modification by those species. We examined the quenching activity of a traditional aldehyde scavenger towards 2-ClHDA. In this experiment, we co-incubated 2-ClHDA with different concentrations of NAC in 2% FBS in DMEM. Figure 3 demonstrates NAC sequesters 80% of 2-ClHDA at a molar ratio of 1/100 (2-ClHDA/NAC). NAC significantly sequestered 2-ClHDA even at a molar ratio of 1/0.5 (2-ClHDA/NAC). 

### 3.4. NAC Reduction of 2-ClFALD in Activated Neutrophils

**Figure 4:**
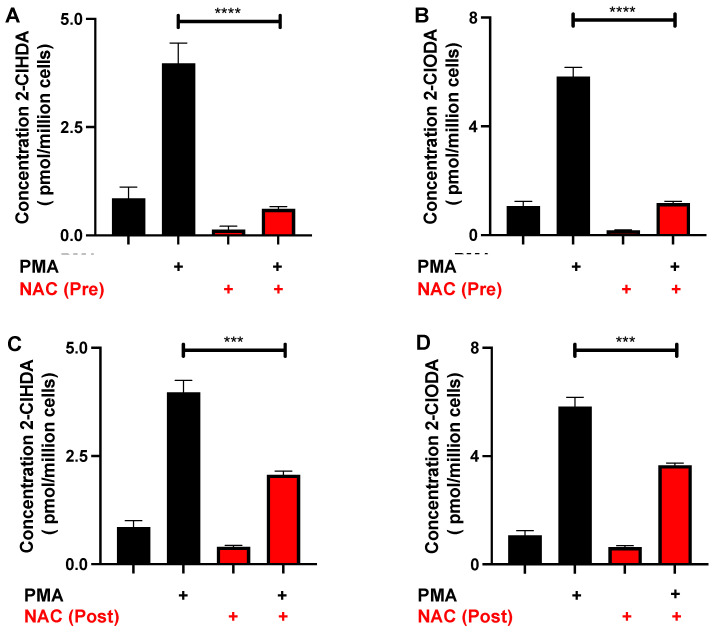
NAC sequesters 2-ClFALD produced by neutrophils. In total, 2 × 10^6^ neutrophils were pretreated (Pre) with 12 mM NAC for 30 min as indicated before activation with 200 nM PMA as indicated (**A**,**B**). In separate experiments, 12 mM NAC was added to neutrophils as indicated 25 min after PMA activation (Post) (**C**,**D**). 2-ClHDA (**A**,**C**) and 2-ClODA (**B**,**D**) molecular species of 2-ClFALD were measured as described in “Materials and Methods”. *n* = 3 for each treatment from independent experiments. *** *p* < 0.001 and **** *p* < 0.0001 for comparison of the amount of 2-ClFALD in PMA-activated neutrophils treated with NAC in comparison to the treatment condition without NAC determined by ANOVA with Tukey’s post-hoc test.

Activated human neutrophils produce 2-ClFALD [3]. Accordingly, the NAC quenching of endogenously produced 2-ClFALD was investigated. A preincubation of neutrophils with 12 mM NAC for 30 min showed a significant decrease in the amount of 2-ClFALD following PMA stimulation (Figure 4A,B). The concentration of the NAC was chosen based on: (1) studies shown in Figure 3 indicating that physiological levels of 2-ClHDA estimated to be as great as 90 μM [3] would require NAC concentrations above 9 mM; and (2) the use of 12 mM and higher levels of NAC used in other cell studies [28,29]. Since NAC also reacts with reactive oxygen species as well as HOCl [30], additional studies were designed to assess the NAC reduction of 2-ClFALD levels following its production in activated neutrophils. Following a 25 min treatment with PMA, subsequent NAC addition reduced 2-ClFALD levels in comparison to conditions with no NAC addition (Figure 4C,D). 

### 3.5. Protective Effects of NAC

**Figure 5:**
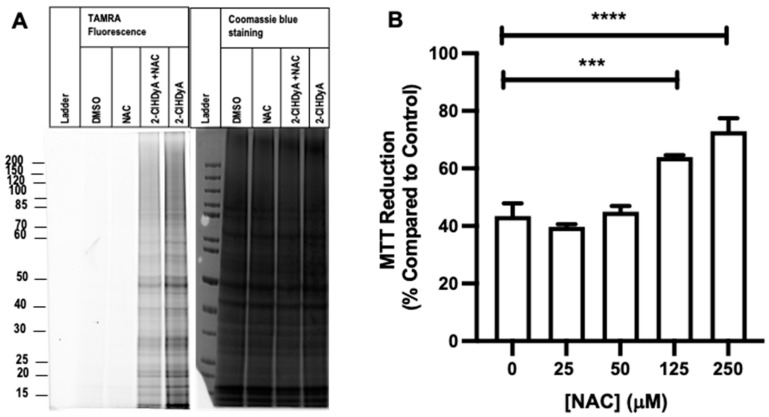
NAC prevents protein modification and toxicity elicited by 2-ClFALD. (**A**) RAW 264.7 cells were pretreated with 250 µM NAC for 30 min at 37 °C followed by treatment with 25 µM 2-ClHDyA for 1 h at 37 °C. Following click reactions with TAMRA-azide, protein fluorescence (TAMRA) and protein (Coomassie blue) were detected. (**B**) RAW 264.7 cells were pretreated with selected concentrations of NAC for 30 min at 37 °C followed by treatment with 25 µM 2-ClHDA for 5 h. MTT assay was performed as described in “Materials and Methods”. *n* = 4 for each treatment from independent experiments. *** *p* < 0.001, **** *p* < 0.0001 for comparison of MTT reduction caused in the presence of NAC preincubation in comparison to the treatment condition without NAC incubation determined by ANOVA with Dunnett’s post-hoc test.

Since NAC sequesters 2-ClHDA, we investigated whether NAC could reduce 2-ClHDA protein modification using the click analog, 2-ClHDyA with RAW 264.7 cells. Protein lysates from cells pretreated with NAC followed by treatment with 2-ClHDyA for 1 h at 37 °C were clicked using a TAMRA conjugated azide. Upon visualization of the TAMRA fluorescence, decreased protein modification was observed in the cells treated with NAC in comparison to the untreated cells (Figure 5A). Since NAC prevented 2-ClHDA interactions with protein, additional studies were performed to investigate whether NAC rescues reduced cell metabolic activity caused by 2-ClHDA. The preincubation of NAC for 30 min significantly prevented the decreased metabolic activity caused by 2-ClHDA (Figure 5B). 

### 3.6. Characterization of 2-ClHDA Adduct with NAC

**Figure 6:**
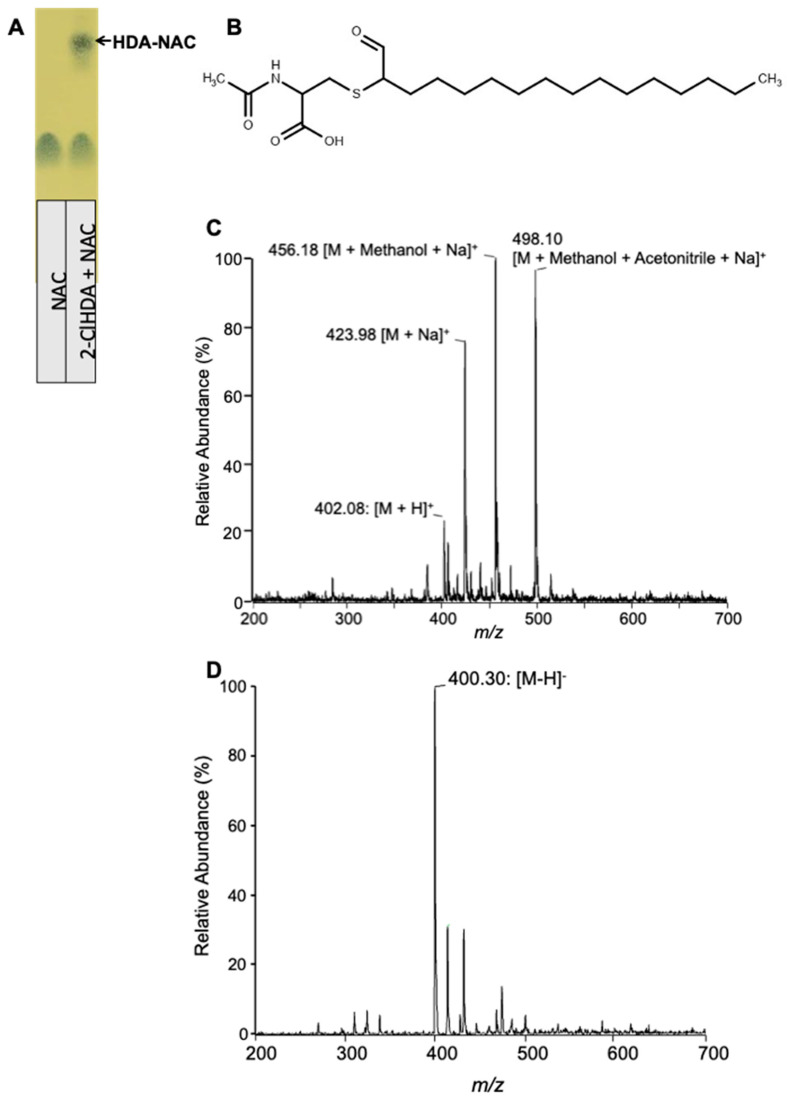
ESI/MS of TLC-purified 2-ClHDA adduct with NAC. The purity of the purified reaction product from 2-ClHDA incubations with NAC was confirmed by TLC and phosphomolybdic acid staining (**A**). (**B**) The putative molecular structure of the adduct HDA–NAC. The purified reaction product was analyzed by a positive ion (**C**) and negative ion (**D**) ESI/MS by direct infusion as described in “Materials and Methods”.

**Figure 7:**
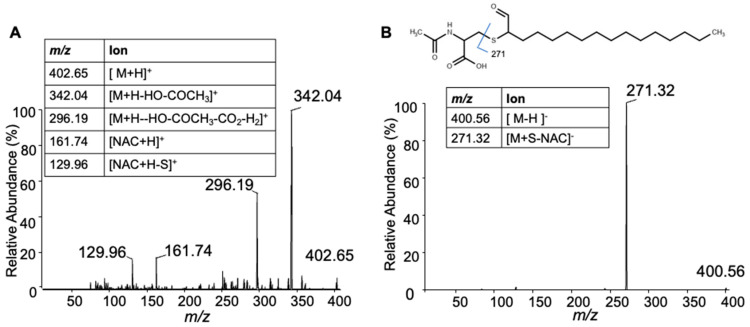
ESI/MS/MS of HDA-NAC. MS/MS spectra for the [M + H]^+^ parent ion at *m/z* 402.65 (**A**) and the [−M − H]^−^ parent ion at *m/z* 400.56 (**B**) are shown. Inset tables provide fragment ion assignments.

Previous studies showed that 2-ClHDA reacts with GSH by a nucleophilic substitution reaction [14]. Here, we examined the reaction of 2-ClHDA with NAC. Initially, 2-ClHDA was reacted with NAC in the molar ratio of 1:10. The reaction products were resolved by TLC and stained with phosphomolybdic acid (Figure 6A). Furthermore, the putative reaction product (Figure 6B) was characterized by ESI/MS/MS. In the positive mode, the reaction product was detected as a molecular ion at *m/z* 402.08 (Figure 6C). Additionally, sodiated, methanol and methanol–acetonitrile adducts of the reaction product were also detected at *m/z* 423.98, 456.18 and 498.10, respectively. The reaction product molecular ions did not show the signature pattern of chlorine-containing molecules (3/1 ratio of *m/z/(m/z + 2)*) indicating a Cl^−^ loss in the reaction. In the negative mode, a major ion was detected at *m/z* 400.30 (Figure 6D). Based on the availability of the carbonyl group and the loss of the chlorine ion, the reaction product is likely formed by a nucleophilic substitution reaction by the sulfhydryl group at the alpha chlorine-containing carbon of 2-ClHDA, resulting in the ejection of the Cl ion (Figure 6B). To further confirm the structure, an MS/MS analysis was performed. The positive protonated precursor ion *m/z* 402.65 fragmented into 342.04 and 296.19. The other two fragments, *m/z* 161.74 and *m/z* 129.96, represent common fragments of NAC (Figure 7A). In the negative mode, the product ion *m/z* 400.56 fragmented with *m/z* 271.32, representing cleavage at the carbon–sulfur bond of NAC as shown in the Figure 7B inset.

### 3.7. Formation of HDA-GSH and HDA–NAC in Neutrophils

**Figure 8:**
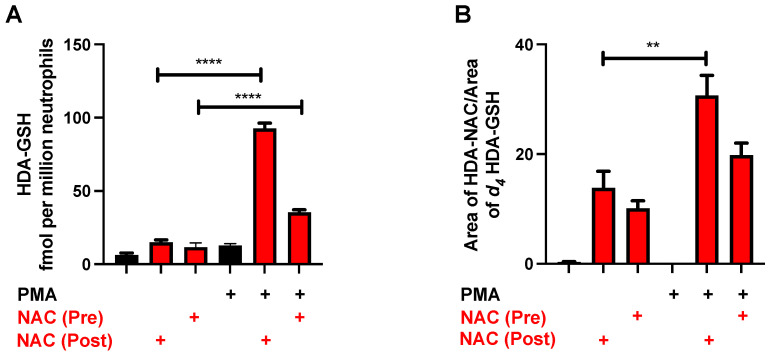
HDA–NAC adduct formation in human neutrophils treated with NAC. In total, 2 × 10^6^ neutrophils were pretreated (Pre) with 12 mM NAC for 30 min as indicated before activation with 200 nM PMA as indicated. In separate experiments, 12 mM NAC was added to neutrophils as indicated 25 min after PMA activation (Post). HDA–GSH (**A**) and HDA–NAC (**B**) were measured as described in “Materials and Methods”. *n* = 3 for each treatment from independent experiments. ** and **** indicate *p* < 0.01 and *p* < 0.0001 for indicated comparisons determined by ANOVA with Tukey’s post-hoc test.

Based on the mass spectrometry characterization of the 2-ClFALD-NAC adduct, we next examined this product in quiescent and PMA-activated neutrophils treated in the presence and absence of NAC. The data in Figure 8 show increases in HDA–GSH and HDA–NAC in neutrophils treated with NAC 25 min after PMA activation in comparison to the neutrophils pretreated with NAC prior to PMA activation. Interestingly, there is more HDA–GSH produced in the neutrophils treated with NAC in comparison to those without NAC treatment (Figure 8A) and significant increases in HDA–NAC are present in quiescent neutrophils treated with NAC compared to cells in the absence of exogenous NAC (Figure 8B).

## 4. Discussion

2-ClFALD is produced when myeloperoxidase-derived HOCl or inhaled chlorine gas-derived HOCl targets the plasmalogen of neutrophils, endothelial cells or epithelial cells [3,9,17]. 2-ClFALD has been shown to accumulate in the lungs of chlorine gas-exposed mice, infarcted rat myocardium and human aorta [4,5,9]. 2-ClFALD has been shown to cause cytotoxicity in several cell lines [10,11,12]. It is likely that the 2-ClFALD modification of proteins is responsible for many of the biological properties of 2-ClFALD. 2-ClFALD is an electrophile that modifies small molecules such as glutathione and proteins by Schiff base adduct formation or alkylation [25]. Thus, the sequestration of 2-ClFALD has the potential to reduce the cytotoxic effects of 2-ClFALD. Here, we show that 2-ClFALD modifies RAW 264.7 cell proteins and reduces metabolic activity. It should be appreciated that the effects of 2-ClFALD may be mediated or modulated by the reactions of 2-ClFALD with free cysteine, small peptides containing cysteine and proteins in the media of these reactions. This reactivity of 2-ClFALD is demonstrated by both protein modification and cell metabolic activity changes elicited by 2-ClFALD being reduced by NAC treatment. Furthermore, the NAC adduct of 2-ClFALD is characterized as an adduct similar to our previous demonstration of 2-ClFALD reactions with GSH [14]. This adduct is also formed in neutrophils treated with NAC, and NAC reduces 2-ClFALD accumulation in activated neutrophils. NAC had the most profound effects on 2-ClFALD when provided after PMA activation. NAC pretreatments consume HOCl production and thus 2-ClFALD is not produced to appreciable levels. Additionally, NAC–HDA adduct formation is much greater under conditions applying NAC after 2-ClFALD production. 

Phloretin has also been examined as a sequesterer of 2-ClFALD in brain endothelial cells [11,12]. Phloretin is a natural polyphenol, a dihydrochalcone, which demonstrates antioxidative, anti-inflammatory, anti-microbial, anti-allergic, anticarcinogenic, anti-thrombotic and hepatoprotective activity [31]. Phloretin is clinically approved for topical use only. The study with phloretin suggests that the sequestration of 2-ClFALD can alleviate its effects. In the present studies, we have examined the benefits of NAC, which is also clinically approved and is mainly used as a mucolytic and an antidote to acetaminophen toxicity. We also chose to investigate NAC as a 2-ClFALD sequesterer since it is a product of the detoxifying mercapturic pathway [32]. Similar to GSH, NAC reacts with the electrophilic carbon with the chloride leaving group, resulting in the alkylation of the NAC sulfur. NAC is also a precursor of GSH [33], and thus NAC conversion to GSH is also a mechanism to reduce 2-ClFALD through GSH-mediated sequestering [14]. Although not determined in these studies, it is also likely that 2-ClFALD reacts with cysteine following the deacetylation of NAC leading to cysteine adducts.

NAC has been previously shown to reduce 2-ClFALD protein modification in THP-1 cells [15]. Similarly, NAC reduced the 2-ClFALD modification of RAW 264.7 cell proteins. Confocal microscopy also showed that 2-ClFALD-modified RAW 264.7 cell proteins were localized in the mitochondria, Golgi and ER. Additionally, the results herein are the first to show that NAC reduces alterations in metabolic activity elicited by 2-ClFALD. Interestingly, NAC has been shown to protect against chlorine gas-induced damage and acute lung injury [34,35]. Since 2-ClFALD is produced in chlorine gas-exposed mice [9], it will be interesting to investigate if the levels of 2-ClFALD in NAC-treated chlorine gas-exposed animals are diminished and examine associations with reduced acute lung injury.

Increases in the plasma levels of the metabolic oxidation product of 2-ClFALD, 2-ClFA, correlate with sepsis mortality in both humans and rodents [6,36]. The presence of 2-ClFA during sepsis indicates that its precursor, 2-ClFALD, is also produced. However, 2-ClFALD is not detected in specimens from septic humans and rodents, which likely reflects either the rapid metabolism of 2-ClFALD or its reactivity with proteins and small molecules. Since NAC has shown protective effects in endotoxemia and sepsis [37,38], it will be of interest in future studies to examine the presence of 2-ClFALD–NAC adducts in septic rodents treated with NAC. 

The studies herein are the first to examine the impact of NAC treatments on endogenously produced 2-ClFALD in human neutrophils and the appearance of HDA–NAC and HDA–GSH. The NAC treatment reduced 2-ClFALD levels following neutrophil activation. This treatment also led to an increase in HDA–NAC, indicating that the sequestration of 2-ClHDA by NAC was responsible for the reduction in 2-ClFALD. Interestingly HDA–NAC was also detected in unstimulated neutrophils suggesting either the basal production of 2-ClFALD or some degree of neutrophil autoactivation under the incubation conditions [39]. HDA–NAC was, however, not detected in neutrophils that were not treated with NAC. Both NAC pretreatment and post-treatment of PMA-stimulated neutrophils led to the increased production of HDA–GSH, indicating that NAC was readily metabolized to GSH.

## 5. Conclusions

2-ClFALD decreases cellular metabolic activity, which may be due to its electrophilic properties leading to protein modification. These studies extend our previous studies showing GSH reactivity with 2-ClFALD by showing for the first time adduct formation with the GSH precursor, NAC. Herein, NAC is shown to reduce 2-ClFALD alterations in cell metabolic activity and protein modification as well as reduce endogenous levels of 2-ClFALD in activated neutrophils with a concomitant production of NAC and GSH adducts of 2-ClFALD.

## Data Availability

All data are within the manuscript.
